# Implanted functional electrical stimulation: case report of a paraplegic patient with complete SCI after 9 years

**DOI:** 10.1186/1743-0003-11-15

**Published:** 2014-02-24

**Authors:** David Guiraud, Christine Azevedo Coste, Mourad Benoussaad, Charles Fattal

**Affiliations:** 1 DEMAR team, LIRMM, Inria, University of Montpellier, 161 Rue Ada, 34095 Montpellier Cedex 5, France; 2 Centre Neurologique mutualiste Propara, Parc Euromedecine, 263 Rue du Caducée, 34090 Montpellier, France

**Keywords:** Spinal cord injury, Paraplegia, Gait, Functional electrical stimulation

## Abstract

**Backgrounds:**

Experience of an implanted functional electrical stimulation neuroprosthesis (FES) associating 8-channel epimysial and 4-channel neural stimulations. The primary objective consisted in presenting clinical and technological experiences based on a 9-year follow-up of one patient implanted with this FES device. The secondary objective consisted in assessing resulting functional benefits.

**Methods:**

One patient recruited in 1996 within the European Stand Up and Walk Project benefited from a 9-year follow-up with clinical and technological evaluations.

**Results:**

The patient was still using the system nine years later making this a unique case, even when compared to other similar studies. The analysis of muscular response to FES underlined the great variability of stimulation thresholds evolution (−26% to +360%, mean +110%) and quality of the induced contraction. Three muscles out of five scored at least 4/5 on the Medical Research Council scale, all stimulated via neural pathways. The patient used the system once a week for 6 years, up to 2006, due to lack of use, the FES-induced muscular response worsened even though the implant was properly functioning, leading to significant decline in gait performances (best 3.45 m/s on 2.9 m), due to muscle fatigue and loss of muscle mass.

**Conclusion:**

Two major issues arise: first the importance of muscle fatigue, underlining the relevance of muscle strength training, and second technological hurdles raising up the question of neural vs. epimysial FES. This advanced technology proves the concept of restoring lower limb motor functions in patients with spinal cord injury. The main features of the stimulation device remain stable even after long periods of inactivity, yet there is a real need for close clinical and technological monitoring.

## Background

As reported in the literature, patients with paraplegia foster the hope of recovering gait and standing abilities [1,2]. If we focus on patients with complete Spinal Cord Injury (SCI), functional electrical stimulation (FES) has been reported as the only technical solution for restoring muscular activity below the level of injury. FES techniques can use surface or transcutaneous electrodes on lower limb muscles. To date, the Parastep® system remains the best illustration of the use of this FES technique, especially regarding its long-term follow-up [3]. As previously reported in [4], transcutaneous electrodes enable FES-assisted gait on the long term (up to 17 years in some studies) with numerous stimulation sites (16 to 26). However, for 2 patients enrolled in the experiment, the main issue was broken electrodes that needed to be changed once a year. Infection remains an issue even though it was controlled in this study.

To date implanted FES devices for gait restoration, have been restricted to experimental concepts with results reported in the literature containing very little follow-up data compared to the Parastep® system. FES approaches vary according to motor activation modalities (i.e. intramuscular, neural, nerve root or spinal cord) and surgical techniques [5-13]. Functional results are mostly restricted to maintaining a standing position, completing a simple step cycle and executing the swing phase for each lower limb with knees locked and the help of a walker. One of the main issues of these studies is the long-term follow-up of the implanted patients. Besides, when devices are prototypes never commercialized, they are used for very small sets of patients. Kobetic et al., 1999 [13] reports a follow-up for more than one year with one patient who used a pattern stimulation similar to what we propose. They show that the patient can walk with a system composed of 16 epimysial channels. Functional results are close to the best ones we obtained but the follow-up is limited to almost one year.

In the framework of the Stand Up and Walk (SUAW) project, started in 1996 as part of the European BIOMED II program, two patients were implanted with an FES neuroprosthesis according to a technique combining epimysial and neural stimulation [14-16]. One of the patients had the system taken out and the case is thus not reported in this paper [15].

The objectives of this work were: i) to present the clinical and technological experience of our team based on a 9-year follow-up, ii) to assess analytic and functional benefits of this FES-implanted system on one patient.

## Methods

### Patient characteristics

The patient was initially selected based on the inclusion criteria described in [15] within the European project SUAW. Medical characteristics are summarized in Table 1.

**Table 1:** Patient characteristics

Sex	Male
Initial Surgery	September 28, 1999
Weight	75 kg
Height	1.75 m
Trauma	Traffic accident
Lesion	T8, AIS A
Spasticity	Modified Ashworth Scale≤1
Spontaneous Reflexes	Penn Scale = 1
Neural channels	Peroneal branch of sciatic nerve
	Femoral nerve (quadriceps)
Epimysial Channels	Gluteus Maximus
	Gluteus Medius
	Iliacus
	Hamstrings
Training, Initial recommendations	3 times a week, 1 hour, from March to July 2000
	1 time a week, 2 hours, from September 2000

### Preoperative procedures

#### Muscle selection and preparation

For each of the patient’s lower limb, six muscles were chosen based on their kinematic contribution to achieve a comfortable standing position and assisted gait [17]. This selection was validated by preoperative surface FES trials (except for the iliacus) and during surgery the team identified motor points and validated the efficacy and selectiveness of neural stimulation: a) *gluteus maximus (GMa)*, *gluteus medius (GMe)*, and *iliacus (Il)* as hip flexors/extensors and stabilizers, b) *quadriceps (QU)* and *biceps femoris ± semi-tendinosus (HA)* as knee flexors-extensors, c) *tibialis anterior (TA)* as dorsiflexors of the ankle.

We used electrical mapping with surface electrodes to test FES-induced muscle strength for all accessible muscles. Only the iliacus could not be electrically stimulated through surface FES. Each muscle underwent 12 weeks of surface FES training achieve maximum contraction level i.e. 4/5 MRC.

### Materials

a) Implanted stimulation generator

It is a current-controlled generator able to provide rectangular pulses followed by a passive exponential recovery phase in order to balance the charge injection, as previously detailed in the literature [14]. Two types of electrodes were used:

•Unipolar epimysial electrodes and two anodes used for a return current path associated with in hemispherical platinum stimulation sites custom-made by IBMT (Franhaufer Institute, St Ingbert, Germany) (hemispherical platinum contact, 8 mm diameter). Intensity ranged from 100 μA to 25.5 mA, 100 μA steps.

•Bipolar neural electrodes (Atrotech Ltd. Hermiankatu 6-8 F 33720 Tampere, Finland) half-cuff electrodes with 2 stimulation sites along the nerve axis. Intensity ranged from 50 μA to 3.15 mA, 50 μA steps.

•Maximum common Frequency 31.25Hz and maximum Pulse width 816 μs by 3.2 μs steps.

b) External control unit

The external control unit includes two processors (PIC™ 16 F873, Intel™ 80C196) [14]. The patient interface consists in four push buttons mounted on the walker, two on each handle and directly connected to the control unit.

c) Software applications

A physiotherapist and a Physical Medicine and Rehabilitation (PM&R) physician made complex parameter adjustments and program changes via the PC interface, while the patient controlled the system [14]. At the time, no closed-loop system was implemented, however this could have easily been achieved with analogue inputs. With the software the physiotherapist was able to launch scenarios, i.e. chains of programmed stimulation sequences, each sequence generating a single movement phase. Thus, a complex action like walking was first decomposed into basic movements: double support or standing, stance phase and swing phase. The transitions between two phases were predefined and could be chained automatically or triggered by the patient, via two push buttons, based on four different actions: a) right (R), b) left (L), c) both buttons pressed (R and L), or d) predefined number of loops (ES). When no action was taken, the ongoing sequence continued to run.

For each sequence, the control parameters enabled intensity or pulse width modulation. The stimulation envelope for each muscle was then defined through a 32-point linear interpolation (minimum time resolution was 0.1 s). During real time execution, each independent stimulation envelope could be modulated through scale adjustments if the physiotherapist had pre-activated this modality. Thus, the patient was able to adapt the stimulation levels of each QU when fatigue occurred through two dedicated push buttons. The second parameter consisted in a set value programmed individually on each channel. Finally, the FES device had a global frequency control fixed at 31.25Hz for home use.

The embedded processors could store and run up to 8 scenarios, each composed of a maximum of 128 sequences: e.g. walking, muscle strengthening and sit-to-stand. This flexible software environment, called “StimManager”, was used to manage the FES device. When at home, the patient was able to perform preset scenarios, but was unable to access the PC interface.

#### Surgical procedure

The complete procedure has already been described in the literature [16]. Motor point locations were determined through continuous stimulation, especially for epimysial electrodes. At the end of the procedure we assessed functional activation of each muscle.

#### Evaluation procedure

The patient went to his local rehabilitation center (Centre Clémenceau, Strasbourg) in 2007 to undergo an evaluation of walking performances through motion capture analysis. The system was used according to the guidelines of the SUAW project so the initial signed inform consent form as well as ethics committee approval (Montpellier, France 1999) were sufficient. Indeed, this reported study was part of the maintenance and follow-up of the patient’s implanted system.

In 2009, we performed a similar evaluation as part of a larger protocol approved by the local ethics committee for which the patient signed an informed consent form (CCPPRB Nîmes, France 2008), we thus were authorized to run additional assessments with an isokinetic chair and evoked EMG recordings [18].

For the present publication of data and figures, the patient read the manuscript and we collected the patient’s signed informed consent form.

The evaluation consisted in an analytical approach of:

**Figure 1:**
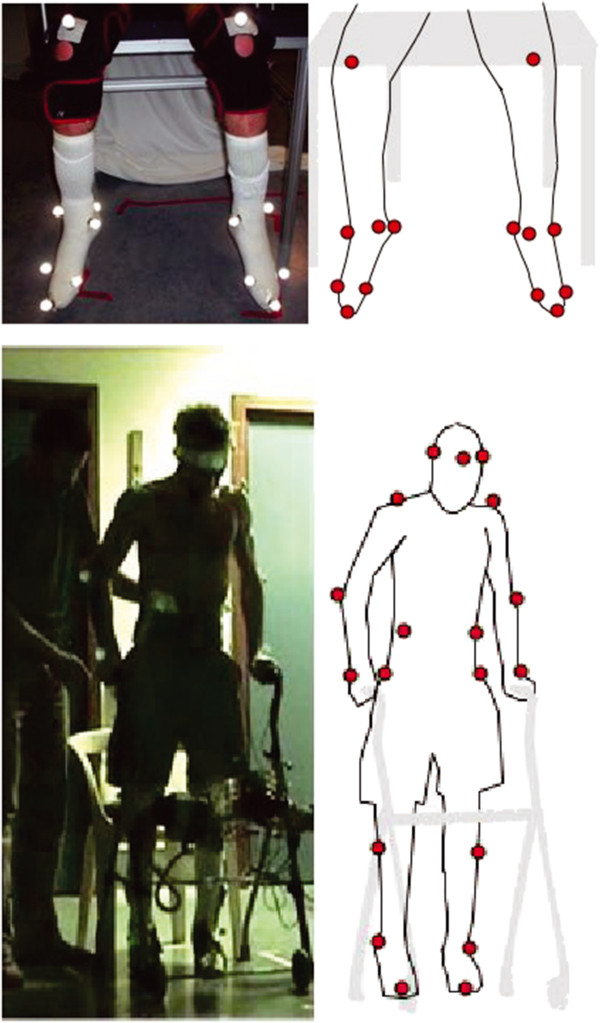
**Marker positions for ankle movement measurements (14 markers, above the knee (should not move used as reference), lateral and medial maleolus, calcaneus, lateral and medial forefoot, tip of 2nd toe) and for gait assessment (19 markers, forehead, ears, shoulders, elbow, wrists, 10th rib, iliac crest, knees, ankles, toes).** We adapted the marker location compared to usual landmarks used for VICON reconstruction in order to cope with practical constraints: use of a walker hiding the legs, trunk bended position during walk, experimenter aid.

**Figure 2:**
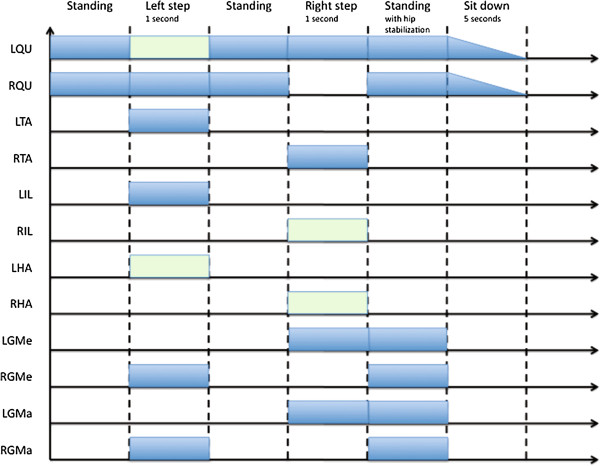
**Sequences of stimulation required for a full step and used for gait assessment.** Stimulation amplitude is not represented because it depends on muscle fatigue and individual features. The green rectangles show facultative muscle contractions that improve the comfort of the patient but not the performances

a) Walking performances

We assessed the gait process using a three-dimensional analysis Vicon 350 system (VICON Motion System Ltd., Oxford UK - 4 cameras, 50 Hz acquisition rate, 19 markers, Figure 1). The following parameters were recorded: step length, step duration, heel lift and gait phases. Stimulation parameters were set to obtain standardized gait, yet the patient could manually and in real- time adjust stimulation levels for both QU according to muscle fatigue. Furthermore, most parameters were set to the maximum at the end of the sessions: i.e. impulsion at 31.25Hz, and width at 600 μs, the intensity depended on the channels used: 25.5 mA on epimysial channels, 3.15 mA on neural channels. Muscle activation sequences are listed in Figure 2.

b) Muscular response to stimulation.

For each muscle the following parameters were recorded by a PM&R physician:

•Intensity threshold levels needed to trigger a muscle contraction.

•Intensity levels to achieve the highest muscle contraction possible on the MRC scale for the QU, TA, HA or intensity levels needed to trigger movement at 4/5 MRC for the Il as well as the GMa and GMe.

•Intensity thresholds responsible for spreading the muscle contraction to the surrounding muscles.

c) Kinematic analysis of the ankle

We used a three-dimensional analysis Vicon 350 system (VICON Motion System Ltd., Oxford UK - 4 cameras, 50 Hz acquisition rate, 14 markers, Figure 1) to explore ankle kinematics during TA stimulation. In fact, since TA contraction is only used during the swing phase, ankle kinematics are essential especially during dorsiflexion. The ankle was not used for balance control in double stance position, so we did not investigate isometric contractions.

d) Torque analysis at knee level

A System 3 Biodex positioning isokinetic chair (Biodex Medical Systems, 20 Ramsay Road, Shirley, New York, USA) was used to measure knee torque when stimulating the QU in isometric conditions at the optimum knee angle around 70° for both legs – deduced from torque-length relationship -. We assessed the QU recruitment curve since stimulation intensity could be modulated for these muscles. Indeed, these muscles generate major torque to counteract the force of gravity during sit-to-stand and at the beginning of the swing phase. They are also involved in the knee locking process. Conversely, HA were only used to reinforce knee locking or slowing down the angular velocity of the knee at the end of the swing phase. They were controlled using an “on-off” activation mode, thus making it useless to evaluate their recruitment curve.

## Results

1) Global results

Proportional control of muscle force, even though possible on all muscles, was never applied on GMa, GMe, Il and HA. Indeed the “on-off” control was enough to have these muscles contribute to posture stabilization. The TA was assessed through the induced kinematics because its role was to promote sufficient dorsiflexion (see below) and the force generated by each QU was modulated by 2 push buttons manually controlled by the patient. Indeed, these muscles were always the first ones to be fatigued and the patient could feel when the knees unlocked. The initial value was set to reach knee lock with the smallest activation level to avoid unnecessary fatigue.

2) Walking performances

One-week post surgery, the patient started a regular muscle strengthening program using the implanted system (Table 1). At the end of program, he was able to stand up and walk for a few steps assisted with a walker.

After a month of intensive training, the patient was able to stand up for 10 minutes. A few additional weeks later his performances leveled-off to 30 minutes of standing up. Furthermore, the patient was able to walk for about 30 minutes total spread-out over five to ten sessions per day, corresponding to a total distance of about 100 m. Average step length was 30 cm [15]. During 6 years, the patient used the system once a week at home.

When we met the patient, he reported that he stopped using the system in 2006. One year later, in December 2007, during one of the planned evaluation, he was still able to walk for approximately 3 meters (Table 2). However, muscular response to stimulation had worsened and there was a quick onset of fatigue limiting gait duration even though the implant was working properly, however gait kinematics remained correct.

In spite of the dissymmetry noted – toe clearance and ratio between swing and support phase -, gait was quite symmetrical as regard other kinematics data (Table 2). We noted a significant improvement (2,81 cm/s to 3,45 cm/s) of the performances between the first and second trial, as evidenced by a greater step length both on the left and right side and shorter step duration.

During the 2009 evaluation, the implanted system did not show any dysfunctions, but the patient was barely able to get up and could not walk. The patient’s muscular capacities had dramatically decreased due to disuse of the system; we decided to focus on assessing the capacity of each individual muscle in order to better quantify this loss of performance.

3) Muscle response

The analysis of muscular response to stimulation is reported in Table 3 and detailed for each muscle, which underlined the great variability of stimulation thresholds and quality of the induced contraction. Testing of proximal and deep muscles is quite difficult in patients with SCI, thus the GMa, GMe and Il muscles could not be quoted on the MRC scale. The HA on the right side did not respond to stimulation. Three muscles out of five scored at least 4/5 on the MRC, all stimulated via a neural pathway.

4) Ankle kinematics

**Figure 3:**
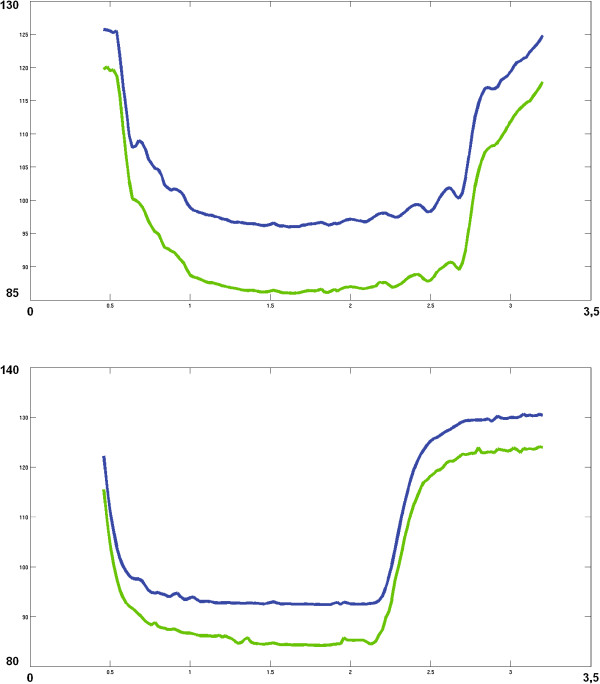
**3D Ankle joint angle (top: right, bottom: left) excursion considering the toe endpoint (green) and orthogonal to metatarsus-phalanx joint (blue) vs. internal and external malleolus.** X-Axis in seconds, Y-Axis in degrees. Stimulation providing the maximum dorsiflexion was programmed (Table 3).

We studied ankle kinematics since they condition proper step passage during the swing phase and thus promote efficient gait dynamics. The kinematic analysis of the ankle showed active differential dorsiflexion motion at 39°±1° for the left ankle and 32.5°±2.5° for the right ankle. In both cases it was well beyond the 10° above the horizontal plane, of dorsiflexion necessary for the swing phase (27° for the left ankle, 20° for the right). The estimated rising time between 10% and 90% of the complete dorsiflexion was 280 ms and 390 ms respectively for the left and right ankle whereas plantar flexion took 330 ms and 530 ms respectively (Figure 3, Table 4). As seen in the Results section, stimulating the TA triggered sufficient ankle dorsiflexion (about 20° on the right leg and 27° on the left one) and amplitude (32.5° on the right side and 39° on the left side) with fast dynamics (few hundreds of milliseconds), validating the effectiveness of neural stimulation. The slight dissymmetry between both ankles values was not due to defective stimulation but rather to improper electrode positioning. On the left ankle, dorsiflexion remained a single-degree-of-freedom movement, whereas on the right side we noted some eversion (Table 3).

5) Knee torque

**Figure 4:**
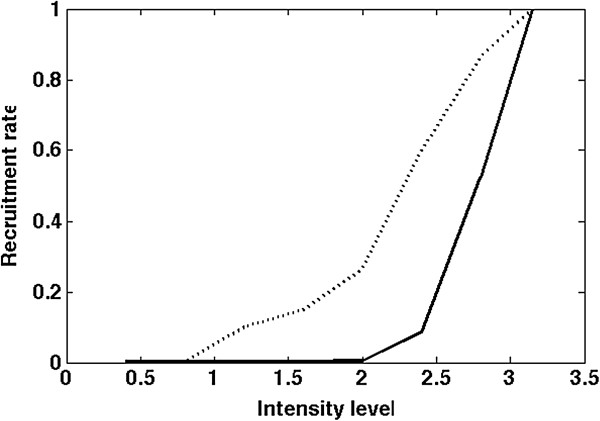
**Recruitment curve of the left QU (solid line) and right QU (dot line) at 25 Hz, pulse width 502.4 μs.** Normalized to the maximum torque obtained at the maximum intensity level (3.15 mA). Intensity varies through 8 steps 0.4, 0.8, 1.2, 1.6, 2, 2.4, 2.8, 3.15 mA with 0.5 s on 0.5 s off to limit fatigue.

Knee torque analysis revealed major dissymmetrical behavior between the right and left QU for the moment and recruiting pattern (Figure 4). The left QU generated a torque 51% higher than the right QU. Control dynamics of the left quadriceps were comprised between 1.1 (threshold detected by physician, 2 mA on the isokinetic chair) and 3.15 mA (maximum ratings) whereas the ones on the right QU were greater, i.e. ranging between 800 μA and 3.15 mA. It implies that control parameters (Intensity) could be more accurately tuned on the right QU to increase the intensity level for torque generation.

**Table 2:** Gait assessment main parameters

	**Trial 01**	**Trial 02**
Total number of steps achieved	21	19
Total distance (m)	2.7	2.9
Total walking duration (s)	95.8	84.1
Step durations		
Left leg mean/std (s)	8.8/2.1	8.1/0.7
Right leg mean/std (s)	8.8/0.5	8.1/0.6
Step length		
Left leg mean/std (cm)	24.1/6.2	28.6/6.9
Right leg mean/std (cm)	25.1/5.3	29.7/4.5
Maximum ankle clearance		
Left leg mean/std (cm)	4.5/1.0	4.2/0.4
Right leg mean/std (cm)	4.2/0.9	3.9/0.7
Maximum toe clearance		
Left leg mean/std (cm)	2.3/0.6	2.8/0.3
Right leg mean/std (cm)	6.0/1.5	4.3/0.3
Ratio for swing/stance phase durations		
Left leg mean/std (%)	43.6/20.4	38.7/7.9
Right leg mean/std (%)	21.0/9.1	24.9/5.6
Ratio for single/double stance durations	44.6/12.1	38.0/7.6

**Table 3:** Muscles main optimal features

**Muscle**	**Thresholds**	**MRC**	**Remarks**
Left TA	1 mA	5 (@2 mA)	Dorsiflexion alone
Right TA	450 μA	5 (@2 mA)	Eversion then dorsiflexion
Left QU	450 μA	4+ (22 N.m) (@3.15 mA, 601.6 μs)	High fatigue, incomplete knee locking, triple reflex may occur
Right QU	1.1 mA	3- (14.5 N.m) (@3.15 mA, 601.6 μs)	Fatigue resistant
Left HA	7 mA	1 (@12 mA)	Triple reflex appears above 12 mA
Right HA	NA		Not responding to stimulation but diffusion occurs
Left GMe	2 mA		Intermittent contraction due to contact problem
Right GMe	8.5 mA	19 mA (saturation)	Nice progressive and selective contraction
Left GMa	11 mA	25.5 mA	Low contraction located on the top of the muscle
Right GMa	1.5 mA	10 mA (saturation)	Nice progressive and selective contraction
Left IL	10 mA	18 mA (optimum)	For higher intensities diffusion to abdominal muscles
Right IL	7.5 mA	20 mA (optimum)	Below some diffusion occurs to abdominal muscles and above to QU

All the measurements, except if noted are performed using a 25Hz frequency and 502.4 μs pulse width, the nominal parameter values. MRC cannot be evaluated for GMe, GMa and IL.

**Table 4:** Kinematics data from ankle joint

	**Left ankle joint**	**Right ankle joint**
Time responses (10%-90%)		
Rising time (ms)	280	390
Falling time (ms)	330	530
Min angle/Max angle		
TEM	84°/124°	86°/121°
MPM	92°/131°	96°/126°
Angle variation		
TEM	40°	35°
MPM	38°	30°
MPM to horizontal correction	−29°	−26°

Toe endpoint (TEM) and orthogonal to metatarsus-phalanx joint (MPM) vs. internal and external malleolus angles are presented.

## Discussion

The 5-year evaluation [15] analyzed thresholds and global gait parameters with stable standing positions and gait durations only. Impedances data could only be retrieved by surgically removing the implant.

When comparing thresholds obtained in [15], we show following evolution versus injected charges (intensity x pulse width) @25Hz, respectively left/right: TA (67%/88%), QU (−16%/360%), GMa (−26%/25%), GMe (158%/137%), Il (319%/79%), HA (17%/NA).

Even though almost thresholds increased, neural stimulation was still more efficient for the resulting functional stimulation (Table 3) with high MRC grades except for the right QU much weakened by disuse. However, for phasic muscles, such as the TA, the contraction remained perfect and useful for more than 10 years even without any specific training. QU stimulation remained efficient even though muscle reconditioning and fatigue had to be addressed via training sessions, but nice contractions were still obtained using stimulation parameters matching the initial system specifications.

Conversely, epimysial stimulation was less stable and failed on the right HA. Furthermore, epimysial stimulation sometimes triggered diffusion or adverse events such as triple reflex probably due to the stimulation of afferent nerve fibers close to the epimysial electrodes. The energy needed was almost 100 times higher so the patient was not as independent when epimysial channels were intensively used.

**Figure 5:**
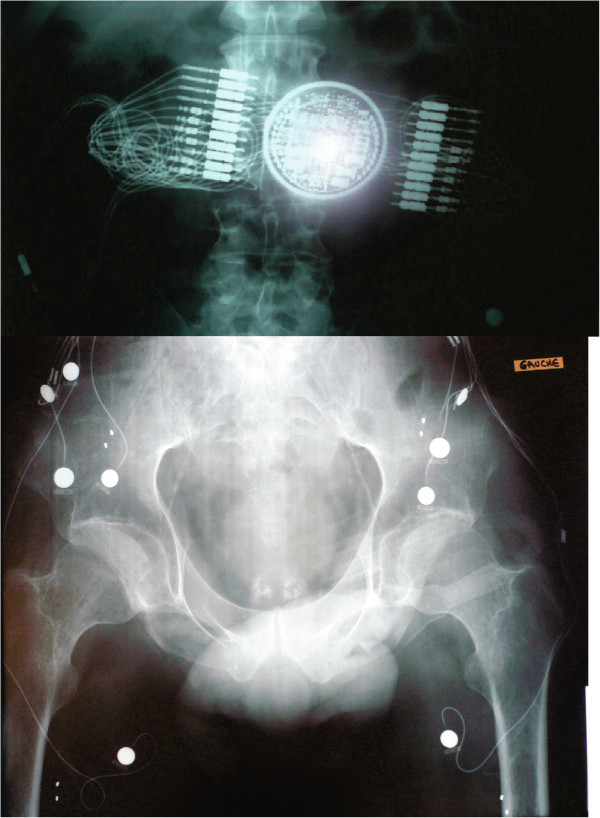
**X-ray (face) of the system obtained in 2009 shows no visible damage of the wires, connectors, electrodes or implant electronics.** Big white dots are epimysial electrodes, small pair of dots are neural electrodes.

The hardware was deemed reliable and we could not determine why right HA stimulation was not possible. This was not due to electronic failure because we observed diffusion among other muscles as well. Indeed, X-Rays (Figure 5) did not evidence faulty electrodes, connectors or wires, or deficient electronic components. Without surgical exploration, we were not able to solve this problem.

### Physical limitations and psychological impact of implanted device systems

For the past 10 years gait orthoses have shown their limits in terms of functional use for movement restoration (while still being useful for rehabilitation purposes). Automated exoskeletons have become more popular in terms of gait possibilities (e.g. Rewalk). However, the issues remain the same and designers do not seem to address them properly. The ergonomics of these biomechanical orthoses or exoskeletons remain incompatible with the patients’ desire to avoid being stared at. Putting on or taking off an orthotic device is still incredibly time consuming. Using a walker to compensate puts an overload on the shoulders with the risk of aggravating rotator cuff lesions. Energy expenditure and cardiovascular solicitations remain quite high for these patients.

Besides, very few studies have reported long-term results on FES-assisted gait restoration for patients with SCI. One reported a 14-month case study with a patient implanted with a 16-channel epimysial system [13]. The walking speed was about 4 times higher than in our study but the patient’s left knee was in a brace. However, during the 2005 evaluation [15] our patient was able to walk almost 100 m a day with comparable step lengths and cadence to the ones reported in [13]. Without exercising the performances decreased a lot. A second long term study reported the results of a percutaneous system on 2 patients after 17 years of use [4]. They report similar distances and step length but higher walking speed, i.e. twice as much as the ones reported in [13]. The system was proved to be quite efficient but they needed to regularly change the electrodes, about once a year, and take special precautions to avoid infections.

We cannot compare more in deep these studies to the present one since no similar measurements were reported.

Today, stimulation offers promising outcomes yet many hurdles still need to be addressed. They are mainly related to muscle fatigue in light of the stimulation intensity proposed. There is a mandatory need for proper vertical alignment of the pelvis, trunk and head, while improving the process of standing up from the wheelchair without causing excessive fatigue. Once these hurdles are addressed and validated, this system could challenge the critics targeted at biomechanical orthoses in terms of ergonomics, energy cost as well as cardiovascular and orthopedic impact.

## Conclusion

This case report shows that the concept of restoring lower-limb motor functions through implanted neural and epimysial stimulations for patients with SCI is possible. The main features of the stimulation remain stable over time even with lengthy disuse periods. However some lessons can be learned from this study: i) neural stimulation may be generalized thanks to the surgical advances witnessed over the past 10 years, indeed a lower current could provide enough energy for an efficient stimulation ii) daily training is essential to maintain muscle trophicity and increase fatigue resistance. We also believe that closed-loop control, in particular for the knee joint may minimize fatigue as the patient did in an empirical manner, provided that angle sensors at the knee joint give the needed feedback measurements. In any case, efficient sit-to-stand movement and balanced standing are expectations most often expressed by the patients [19]. Thus, future studies should focus on developing more efficient neural stimulation devices that could, eventually, control sit-to-stand and balanced standing. The actual implanted patient represents a unique case study.

Furthermore, this case report underlines the importance of closely monitoring the patient both on clinical and technological levels. This monitoring is essential for different reasons: i) assess the patient performances over time, ii) check proper system functioning and provide regular technical updates, iii) learn from the long-term use of such rare neuroprostheses.

## Abbreviations

AIS: American Impairment Scale from the American Spinal Cord Association; MRC: Medical Research Council grading muscle strength from 0 (no movement) to 5 (Normal movement against gravity and against imposed resistance); MAS: Modified Ashworth Scale grades spasticity through 6 levels.; PS: Penn Scale grades the spontaneous reflexes.

## Competing interests

The authors declare that they have no competing interests.

## Authors’ contributions

CF participated in the medical evaluation, drafting the manuscript, revising the manuscript and given final approval for publications. DG participated in the design and development of the FES implanted neuroprosthesis software, data processing as well as drafting the manuscript. CA participated in the biomechanical evaluation and drafting the manuscript. MB participated in the collection of the data, setup of the protocol with the patient and data processing. All authors read and approved the final manuscript.
